# Entry into the lytic cycle exposes EBV-infected cells to NK cell killing via upregulation of the MICB ligand for NKG2D and activation of the CD56^bright^ and NKG2A^+^KIR^+^CD56^dim^ subsets

**DOI:** 10.3389/fimmu.2024.1467304

**Published:** 2024-11-29

**Authors:** Maria Giovanna Desimio, Daniela Angela Covino, Caterina Cancrini, Margherita Doria

**Affiliations:** ^1^ Research Unit of Primary Immunodeficiency, Bambino Gesù Children’s Hospital, IRCCS, Rome, Italy; ^2^ Department of Systems Medicine, University of Rome “Tor Vergata”, Rome, Italy

**Keywords:** Epstein-Barr virus (EBV), lymphoblastoid cell line (LCL), latency, lytic replication, natural killer (NK) cells, cytotoxicity, NKG2D, NKG2A

## Abstract

The Epstein–Barr virus (EBV) is usually acquired during infancy as an asymptomatic infection and persists throughout life in a latent state under the control of the host immune system. However, EBV is associated with various malignant diseases that preferentially develop in immunodeficient individuals. Accumulating evidence suggests an important role for NK cells, though the mechanisms by which EBV evades or triggers NK cell responses are poorly understood. Here, we generated EBV-immortalized lymphoblastoid cell lines stably expressing an inducible form of the BZLF1 early lytic viral protein (LCL-Z) to challenge primary NK cells with EBV^+^ targets in either the latent or lytic phase of infection. We show that entry into the lytic phase results in drastic downregulation of HLA-E but not HLA-A, -B, and -C molecules and in increased expression of ligands for the activating NKG2D receptor, with MICB being upregulated at the cell membrane and released in a soluble form while ULBP2 and ULBP4 accumulate intracellularly. Furthermore, LCL-Z cells are killed by NK cells in an NKG2D-dependent manner and to a much higher extent during the lytic phase, but HLA-class I molecules constrain killing throughout the viral life cycle; unexpectedly, the antibody-mediated block of the inhibitory NKG2A receptor results in reduced lysis of lytic LCL-Z cells that are nearly devoid of the cognate HLA-E ligand. Accordingly, we show that NKG2A^+^ NK cell subsets, specifically CD56^bright^ and NKG2A^+^KIR^+^CD56^dim^ cells, are those that preferentially respond against cells with lytic EBV replication. Overall, these results shed light on NK/EBV^+^ cell interactions providing new information for improving NK cell-based immunotherapies to treat EBV-induced diseases.

## 1 Introduction

Over 95% of the world’s population is infected by the Epstein–Barr virus (EBV), an oncogenic γ-herpesvirus that is transmitted via saliva to naïve B cells in oral lymphoid tissues (1). EBV establishes a latent infection through stepwise restriction of viral protein and non-coding RNA (ncRNA) expression (from type III to 0 latency programs), hence persisting for life in memory B cells in which it may occasionally switch to a lytic replication phase, allowing virus reseeding and transmission to a different host (1, 2). EBV transition from latency to lytic replication is under the control of cellular factors, including signaling pathways, epigenetic regulation, and stress conditions, and of viral gene products, with the immediate-early BZLF1 protein having a key role in the transactivation of most lytic genes that allow the virus to replicate (3). Primary EBV infection normally occurs during infancy without causing symptoms, yet it may lead to infectious mononucleosis (IM), particularly when deferred into puberty; additionally, mostly in individuals with an immunodeficiency caused by co-infections, inborn error of immunity (IEI), or immunosuppressive therapies, EBV is associated with various lymphoproliferative disorders (LPDs) and malignancies affecting B cells or other atypical viral targets, including Burkitt’s lymphoma (BL), Hodgkin’s lymphoma (HL), NK or T cell lymphoma, nasopharyngeal carcinoma (NPC), and gastric carcinoma (GC), overall being responsible for approximately 1.5% of all human tumors (4). EBV-related pathologies arise from impaired or dysregulated immune responses against EBV, from the activity of various viral proteins and ncRNAs that promote tumorigenic transformation and immune evasion, and, possibly, from viral and host factors not yet identified (1, 4–6).

In healthy individuals, EBV-specific CD8 T cells are crucial for viral load suppression during primary infection, then persist as long-lasting memory cells (5). Before development of virus-specific T-cell responses, EBV is challenged by the encounter with innate immune cells, such as natural killer (NK) cells, which are abundant at the site of virus entry and function as the first line of defense against pathogens (7). NK cells are cytotoxic lymphocytes armed with the antigen-independent capacity to kill infected, tumor-transformed, and, in general, “stressed” cells via secretion of lytic granules; such innate response is controlled via the balance between opposite signaling pathways: on the one hand, a negative regulation is exerted by inhibitory receptors, including inhibitory killer immunoglobulin receptors (iKIRs), NKG2A, and LILRB-1, upon engagement with ligands belonging to the HLA-class I protein family; on the other hand, activating receptors, which comprise NKG2D, DNAM-1, natural cytotoxicity receptors (NCRs: NKp30, NKp44, and NKp46), NKG2C, and activating KIRs (aKIRs), mediate NK cell activation following recognition of ligands that are either virally encoded or upregulated on infected/stressed cells (8–10). NK cell-mediated killing also occurs via antibody-dependent cellular cytotoxicity (ADCC) triggered by the CD16 low-affinity Fc receptor or via apoptosis induced by death receptor ligands (TRAIL and CD95-L). Moreover, activated NK cells secrete cytokines, particularly IFN-γ and TNF, and chemokines that attract and stimulate T cells and innate cells, thus promoting broad immune responses (8, 10). Most receptors are expressed in NK cells in a random manner, generating a remarkable phenotypic and functional NK cell heterogeneity (11). In peripheral blood, two NK cell populations can be distinguished: a minor subset (~10%) consists of CD56^bright^CD16^−/+^ cells (referred to as CD56^bright^), which are efficient cytokine producers and commonly considered as immature precursors of the most abundant highly cytotoxic CD56^dim^CD16^+^ subset (referred to as CD56^dim^) (12). Phenotypically, CD56^bright^ cells are characterized by the expression of NKG2A and various activating receptors, while the CD56^dim^ subset comprises a heterogeneous population of NK cells with different degrees of differentiation, which implies the gradual loss of NKG2A and sequential acquisition of iKIRs and the CD57 maturation marker (13, 14). Additionally, among CD56^dim^ cells, a population of NKG2C^+^ cells with features of adaptive immunity, also referred to as Memory-Like, has been identified in HCMV-seropositive individuals (15).

A large body of clinical and experimental evidence supports the important role of NK cells in the control of EBV during primary infection and beyond, as we recently reviewed (16). First, discovery of six isolated NK cell deficiencies as well as impaired NK cell development or function in more than 50 different IEIs illustrated the critical role of these cells in anti-viral defense, particularly in the prevention of severe infection with EBV and other herpesviruses (17). Second, reduced NK cell frequency and/or cytotoxicity were associated with EBV^+^ LPDs and tumors by comparison with equivalent EBV^−^ diseases, pointing towards a key role of NK cells in restraining EBV-related pathogenesis (18–22). Furthermore, studies on acute EBV infection in IM patients and in a humanized mouse model of IM showed the expansion of early differentiated CD56^dim^NKG2A^+^ NK cells with effective anti-EBV activity before initiation of virus specific T-cell responses (23, 24); additionally, NK cell depletion experiments in mice demonstrated that NK cells not only suppressed EBV viremia and prevented EBV-induced malignancies, but also reduced lymphocytosis and the consequent IM symptoms by cytotoxic clearance of activated CD8 T cells (23). Finally, studies in EBV-infected individuals pointed at the anti-viral potential of distinct NK cell subsets, specifically mature CD56^dim^NKG2A^+^CD57^+^ cells (25) and tonsillar CD56^bright^IFN^high^ cells (7).

The knowledge on NK cell biology has greatly increased in recent years, leading to the development of novel immunotherapies such as adoptive transfer of potentiated NK cells, which offer exciting promise for the treatment of EBV-associated diseases. However, for successful clinical applications, current understanding of the mechanisms that regulate the capacity of NK cells to kill EBV^+^ cell targets should be improved. Indeed, several experimental studies have shown the capacity of various EBV proteins and ncRNAs to modulate the expression of ligands for both inhibitory and activating NK receptors, though results are often controversial and little is known on the consequences for NK cell-mediated killing [reviewed in Ref (16).]. As for HLA-class I molecules, these are highly expressed on latently EBV-infected cells through the activity of viral factors such as LMP1 and then they are downregulated by various lytic proteins acting at different levels (from gene transcription to protein trafficking) once the virus has entered the productive replication program; hence, the interaction of HLA-class I on EBV^+^ cells with cognate iKIRs inhibiting NK cell activation could be either efficient or impaired depending on the latent or lytic phase, respectively. This model is in line with NK cell-mediated killing being more efficient against targets with lytic EBV replication reported in studies *in vitro* and in a mouse model (23, 24, 26), yet is at odds with the lack of KIR expression on those NK cell subsets that react against EBV *in vivo* during IM and upon *in vitro* challenge with EBV-infected targets (24). Another controversial matter concerns EBV modulation of ligands for NK cell activating receptors, particularly those recognized by NKG2D (MICA/MICB and ULBP1–6 family of proteins, referred to as NKG2DLs) and DNAM-1 (CD112 and CD155 proteins, referred to as DNAM-1Ls). Expression of cell-surface NKG2DLs, which is normally highly restricted but can be induced in response to infection, transformation, or other cellular stress (27, 28), was described on latently EBV-infected cells in some reports (24, 29, 30), even though there is no consensus on the type of ligand expressed (i.e., MICA, MICB, ULBP1, or ULBP4) and, in other studies, none of the NKG2DLs was found (26, 31); a few studies also analyzed NKG2DLs after entry in the EBV lytic phase, showing disparate results ranging from further MICA up-modulation (24), *de novo* induced ULBP1 expression (26), or complete absence of NKG2DLs (31). Analogously, contrasting evidence was provided for DNAM-1Ls that, depending on the report, were either absent in all phases of the EBV life cycle (31) or induced during lytic replication (24, 26). Furthermore, experimental studies demonstrated the capacity of individual EBV proteins or ncRNAs to either repress (e.g., mir-BART2/7, LMP2, and EBNA1) or induce (e.g., LMP1 and BZLF1) specific NKG2DL expression, without an apparent separation of inhibitory and stimulatory activities relative to the viral replication phases [reviewed in Ref (16).]. Overall, a growing number of disparate lines of evidence suggest that EBV has evolved strategies to modulate the expression of ligands for NK cell receptors but further investigation is clearly needed to understand which receptor/ligand axes effectively mediate the capacity of NK cells to control EBV infection throughout the viral life cycle.

Here, we set up EBV-immortalized lymphoblastoid cell lines (LCLs) with stable expression of an inducible form of BZLF1 to challenge primary NK cells with targets in either the latent or lytic phase of EBV infection, and investigated killing efficiency, NK cell receptor/ligand interactions, and which subset of NK cells was most responsive.

## 2 Materials and methods

### 2.1 Cells

Primary NK cells and EBV-immortalized LCLs were maintained in complete RPMI 1640 medium (cRPMI) and 293T cells were maintained in complete Dulbecco’s modified Eagle’s medium (cDMEM) both supplemented with 10% fetal bovine serum, 2 mM L-glutamine, and 100 units/mL penicillin–streptomycin (all from Euroclone). PBMCs of healthy subjects were obtained by separation on Ficoll gradients of buffy coats from a donor bank. Ethical committee approval and written informed consent from all participants were obtained, in accordance with the Declaration of Helsinki. Primary NK cells were isolated from PBMCs by negative selection with the EasySep NK-cell Enrichment Kit (Stem Cell Technologies) according to the manufacturer’s protocol. The purity (~95%) of isolated NK (CD3^-^CD56^+^CD16^−/+^) was assessed by immunolabeling and flow cytometry analysis. LCLs were generated by infecting PBMCs with the laboratory strain B95-8 (ATCC) of EBV according to standard techniques, and maintained at ~0.5 × 10^6^/mL by passaging every 4 days and adding fresh cRPMI once a week. Stably transduced LCL-Z cells were maintained in cRPMI supplemented with 1 μg/mL of puromycin (Sigma-Aldrich/Merck) and, to induce EBV lytic replication, 200 nM of 4-hydroxytamoxifen (HT) diluted in cRPMI from a 25 mM stock in methanol (Sigma-Aldrich/Merck).

### 2.2 Generation of LCL-Z cell lines

A lentivirus was generated by a standard protocol based on three DNA vectors including the p8272 plasmid, a kind gift of Paul J. Farrell (Imperial College Faculty of Medicine, London) that codes for the early lytic EBV protein BZLF1 fused to the HT-responsive mutated estrogen receptor hormone-binding domain and for the puromycin resistance enzyme (32), the packaging (psPAX2), and envelope-encoding (pVSV-G) plasmids, which were transfected into 293T cells using Lipofectamine™ 3000 (Thermo Fisher Scientific) according to the manufacturer’s instructions. Forty-eight hours after transfection, the lentivirus-containing medium was harvested, frozen in aliquots and titrated by serial dilution in 293T cell cultures followed by flow cytometry analysis of BZLF1^+^ cells after 2 days. Finally, LCLs were subjected to spin infection [2,000*g* for 60 min at room temperature in the presence 8 μg/mL polybrene (Sigma-Aldrich/Merck)] with lentivirus at a multiplicity of infection (m.o.i.) of 25, then cultivated for 24 h before replacing the medium with fresh cRPMI. Three days later, 1 μg/mL of puromycin was added to cultures to select stably transduced cell lines, referred to as LCL-Z cells, expressing the inducible BZLF1-HT protein.

### 2.3 Measurement of EBV replication by qPCR

To measure EBV replication, total DNA was extracted from LCL-Z cells treated or not with 200 nM HT for 24, 48, and 72 h using the QIAmp DNA Blood Kit (Quiagen) and analyzed by qPCR using SensiFAST SYBR Green PCR master mix (Bioline) using primers specific for the viral *BALF5* gene and the *PIK3R1* control gene (BALF5/F, 5′-CGGAGTTGTTATCAAAGAGGC-3′; BALF5/R, 5′-CGAGAAAGACGGAGATGGC-3′; PIK3R1/F, 5′-TTATCAAGCTCGTGGAAGCC-3′; PIK2R1/R, 5′-TGTAAACGGCTGCTGGAAT-3′). The cycling conditions were as follows: 95°C for 2 min, followed by 40 cycles of 95°C for 5 s and 60°C for 30 s. Quantitative PCR was performed using the Applied Biosystems 7300 Real-Time PCR System. The increase of EBV replication initiated by HT-activated BZLF1 over spontaneous replication was evaluated by dividing BALF5 DNA amounts by those measured in non-treated control samples (hence set to 1).

### 2.4 Flow cytometry

In all experiments except the cytotoxicity assays, cell viability was assessed by using the LIVE/DEAD fixable NEAR-IR dead cell stain kit according to the manufacturer’s protocol (Thermo Fisher Scientific). To label cell-surface molecules, cells were incubated for 20 min at 4°C with specific mouse monoclonal antibodies (mAbs). To detect intracellular molecules, cells were fixed with 1% paraformaldehyde (PFA) for 10 min, then permeabilized with Permeabilizing Solution 2 (BD Pharmingen) for 10 min before incubation at room temperature for 30 min with specific mAbs. For unconjugated mAbs, a further incubation with AF647-coniugated goat anti-mouse IgG (GAM) (Thermo Fisher Scientific) was performed. Immunolabeled cells resuspended in 1% PFA were acquired on a Cytoflex (Beckman Coulter). Positive cell gating was set using fluorescence minus one control (FMO). The specific mean fluorescence intensity (MFI) for each labeled marker was calculated by subtrac8ng the value obtained with isotype control antibody. Data analyses were performed using Kaluza (Beckman Coulter).

The following mAbs were used: CD3/AF700 (UCHT1), CD56/PerCpCy5.5 (B159), and CD16/BV510 (3G8) from BD Pharmingen; NKG2A/FITC and NKG2C/PE from Miltenyi Biotec; HLA-E (3D12) and CD57/PE-Cy7 (TB01) from eBioscience; CD16/AF700 (3G8), NKG2D/BV785 (1D11), KIR2DL1/S1/S3/S5/APC (HP-MA4), KIR2DL2/L3/S2/APC (DX27), KIR3DL1/APC (DX9), CD155 (SKII.4), CD112 (TX313), and CD48/APC (BJ40) from BioLegend; MICA (AMO1) and ULBP3 (CUMO3) from BamOmaB; MICB (MAB1599), ULBP1 (MAB1380), and ULBP2/5/6 (MAB1298) from R&D Systems; and ULBP4 (6E6) and BZLF1/AF488 (BZ1) from Santa Cruz Biotechnology; HLA-A, -B, and -C (W6/32) were kindly provided by Patrizio Giacomini (Istituto Regina Elena, Rome, Italy).

### 2.5 Enzyme-linked immunosorbent assay

LCL-Z cells were plated at 2.5 × 10^6^/mL in cRPMI alone or containing 200 nM HT and exposed 25 μM MMPI-III (Calbiochem/Merck) or equivalent amounts of its solvent (DMSO). After 48 h, culture medium was collected and analyzed by enzyme-linked immunosorbent assay (ELISA) to measure the concentration of sMICA and sMICB (from R&D Systems) according to the manufacturer’s instructions and sULBP2 following a previously described procedure (33).

### 2.6 NK cell lysis assay

Flow cytometry-based cytotoxicity assays were performed using LCL-Z cells treated with HT for 24, 48, and 72 h as targets and purified NK cells pre-activated for 18 h with 500 IU/mL IL-2 (Peprotech) as effectors. Briefly, LCL-Z cells were labeled with Cell Proliferation Dye eFluor™ 450 (eBioscience, Thermo Fisher Scientific) according to the manufacturer’s instructions, washed twice, and then seeded in cRPMI either alone (spontaneous lysis control) or with effector cells at an effector-to-target cell (E:T) ratio of 2:1 for 4 h. Cells in the co-culture were collected and labeled with 7-aminoactinomycin D (7-AAD; Sigma-Aldrich/Merck) for 20 min, fixed/permeabilized, stained for intracellular BZLF1, and analyzed by flow cytometry. The percentage of specific lysis of target cells (gated as eFluor450^+^) was calculated as follows: 100 × (%7-AAD^+^ target cells in sample − basal %7-AAD^+^ target cells)/(100 − basal %7-AAD^+^ target cells). In blocking experiments, prior to co-culture with targets, NK cells were incubated for 30 min at room temperature with saturating amounts of blocking antibodies anti-NKG2D (1D11; R&D Systems) and anti-DNAM-1 (11A8; BioLegend), both used at 1 μg/10^6^ cells, anti-NKG2A (Z199; Beckman Coulter) used at 3 μg/10^6^ cells, or equivalent amounts of purified IgG as control. To block HLA class I molecules, saturating amounts of anti-HLA-class I IgM clone A6136 (1:20 dilution of the A6136 hybridoma supernatant; kindly provided by Lorenzo Moretta, Bambino Gesù Children’s Hospital, Rome, Italy) were added to the medium of the 4-h co-cultures.

### 2.7 Degranulation of NK cell subsets

Effectors and targets were prepared as for the lysis assay; specifically, IL-2-stimulated NK cells were seeded either alone (no target control) or together with targets (eFluor450-labeled LCL-Z cells pre-exposed or not to HT for 48 h) at an E:T ratio of 2:1 in cRPMI. The medium was supplemented with a 1:200 dilution of either anti-CD107a/BV605 or control mouse IgG_1_/BV605 (BD Biosciences). Cells were cultivated for 6 h, with the addition of Golgi Stop (1:1500; BD Biosciences) and Brefeldin A (10 μg/mL; Sigma-Aldrich/Merck) after the first hour, then subjected to staining for cell-surface NK markers (i.e., CD16, CD56, NKG2A/C/D, CD57, NKG2A, and KIR2DL1/2DL2/3DL1) and analyzed by flow cytometry to identify maturation subsets and their degranulation efficiency (i.e., %CD107a^+^) within gated (eFluor450^-^) NK cells.

### 2.8 Statistical analysis

All experiments have been performed independently at least three times. GraphPad Prism 6.0 software was used to perform all statistical analyses. A value of *p* < 0.05 was considered statistically significant.

## 3 Results

### 3.1 Activation of inducible BZLF1 efficiently initiates lytic EBV replication in transduced LCLs

**Figure 1:**
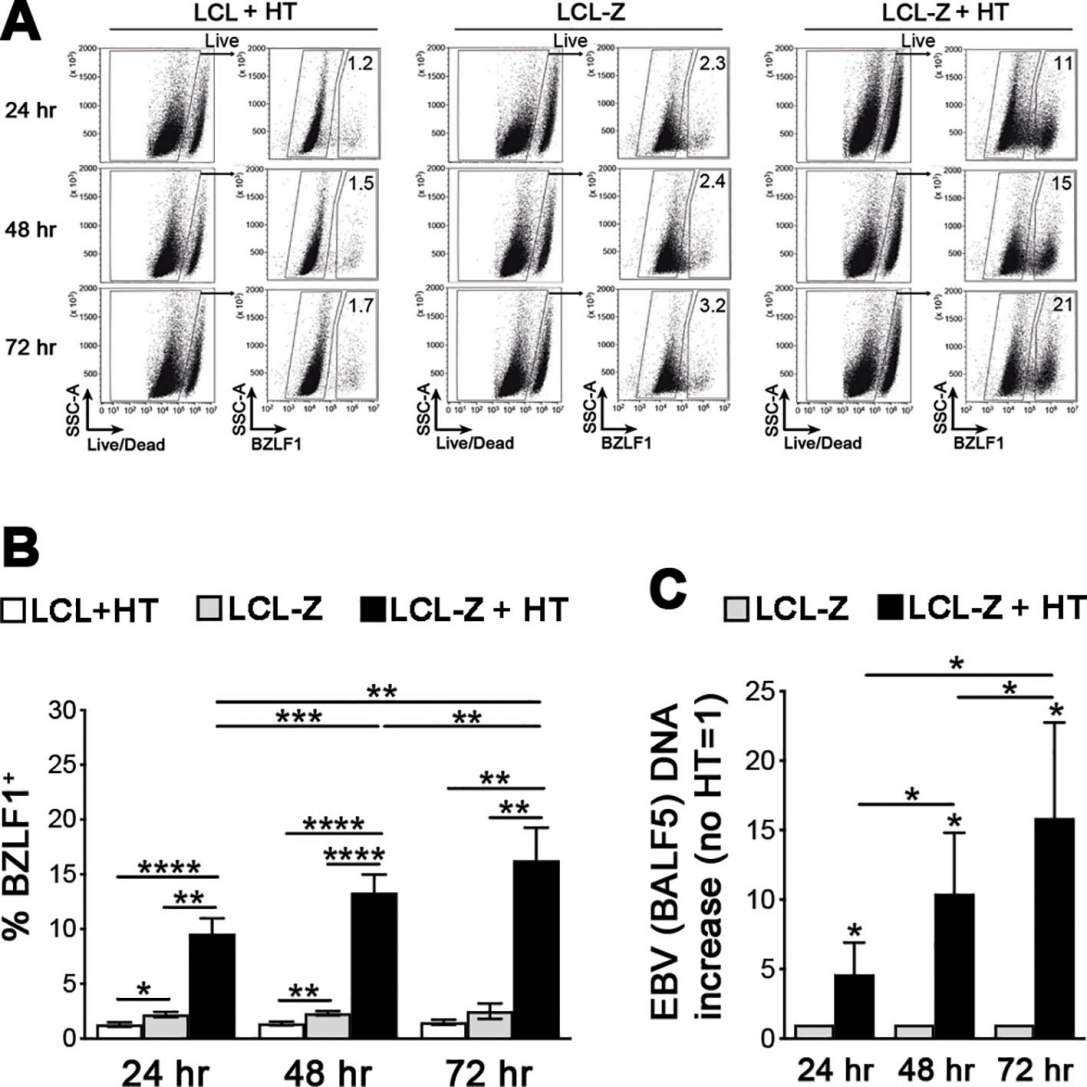
BZLF1 activation triggers efficient EBV lytic replication in LCL-Z cells. **(A)** Representative flow cytometry analysis of BZLF1 expression in LCL-Z cells after culture with or without 400 nM HT for 24, 48, and 72 h; original non-transduced cells treated with HT (LCL + HT) are also shown. **(B)** Bar graphs with dot plots show the mean ± SEM percentage of BZLF1^+^ cells in LCLs derived from six different donors that were stably transduced with BZLF1 and exposed or not to HT for 24, 48, and 72 h (LCL-Z and LCL-Z + HT, respectively); in parallel, non-transduced LCL-Z cells were exposed to HT and analyzed. **(C)** LCL-Z cells treated or not with HT for the indicated time were subjected to qPCR to quantify EBV viral DNA. Each bar represents the mean and standard deviation for the viral DNA level (e.g., *BALF5* polymerase gene) after normalization, calculated by dividing for the DNA level in the untreated sample for six independent experiments. *p < 0.05, **p < 0.01, ***p<0.001, ****p<0.0001 by paired *t*-test.

Very useful for experimental studies is the capacity of EBV, specifically the B95-8 prototype strain, to transform *in vitro* primary B cells into LCLs that express the full latency program (type III: six EBNA proteins, LMP1, LMP2, and various ncRNAs) and grow indefinitely in culture. Here, to study NK cell interactions with EBV-infected cells in both latent and lytic phases of the virus life cycle, we generated healthy donor-derived LCLs with constitutive expression of the immediate-early lytic EBV protein BZLF1 that is kept inactive by fusion to an HT-responsive portion of the estrogen receptor, herein referred to as LCL-Z cells. As measured by flow cytometry in a 72-h time course, the low frequency of BZLF1^+^ cells that spontaneously enter the lytic phase in LCL-Z cultures rapidly rises and increases over time upon the addition of HT (**Figures 1A, B**), in line with BZLF1 functioning as the key switch between latent and lytic replication. As expected, non-transduced LCL cultures do not respond to HT treatment and present a very low percentage of BZLF1^+^ cells analogously to untreated LCL-Z cultures (**Figure 1B**). The increment of lytic EBV replication was confirmed by measuring viral DNA that increased in LCL-Z cells upon HT exposure for 24, 48, and 72 h by 5-, 10-, and 15-folds, respectively, as compared to untreated cells (**Figure 1C**).

### 3.2 The switch to lytic EBV replication induces the expression of MICB and downregulates HLA class I molecules, particularly HLA-E

**Figure 2:**
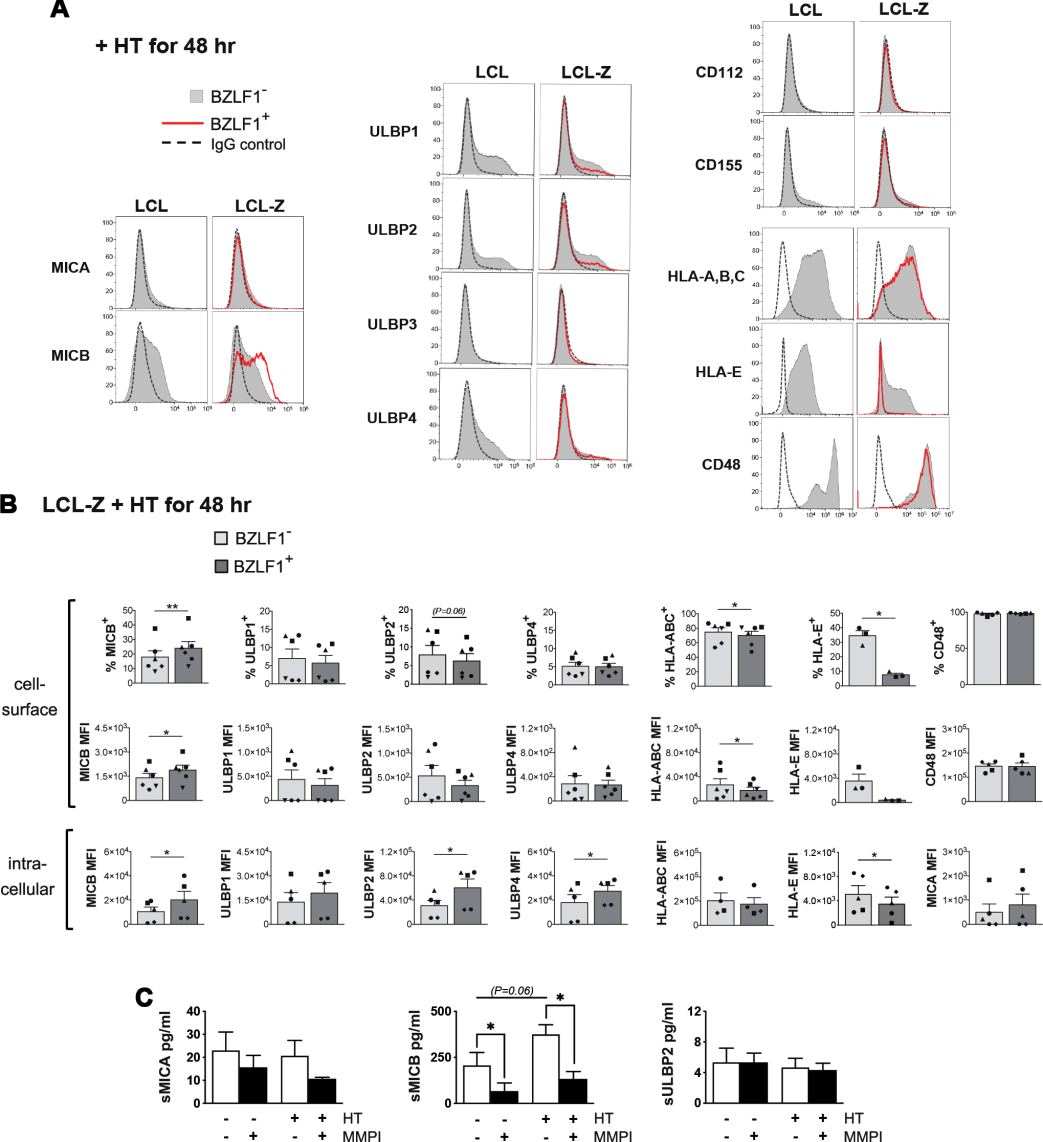
Expression of ligands for NK receptors during the latent and lytic phases of the EBV life cycle. **(A)** Flow cytometry histograms comparing the levels of NKG2DLs (MICA, MICB, and ULBP1–4), DNAM-1Ls (CD112 and CD155), HLA-A, -B, and -C, HLA-E, and CD48 on gated BZLF1^+^ (red line) and BZLF1^−^ (filled gray) cells in LCL and LCL-Z cultures treated with HT for 48 h are shown for a representative experiment. Staining with control Ig isotype is depicted as a dashed line. **(B)** Bar graphs with dot plots depict the mean ± SEM percentage of positive cells and mean fluorescence intensity (MFI) at the cell surface (top panels) and MFI obtained by intracellular staining (bottom panels) for NK receptor ligands expressed in BZLF1^−^ and BZLF1^+^ LCL-Z cells exposed to HT for 48 **(h)** Five or six different LCL-Z cells (each represented with a distinctive symbol) were analyzed for each molecule. Comparisons between BZLF1^−^ and BZLF1^+^ cells were performed by paired *t*-test. **(C)** Soluble NKG2DLs (sMICA, sMICB, and sULBP2) were measured in the medium of LCL-Z cells cultivated for 48 h without treatments or in the presence of HT and/or MMPI. Bars show mean ± SEM from three independent experiments and statistics was performed using paired *t*-test. Significant differences are indicated (*p < 0.05, **p < 0.01) as well as nearly significant *p*-values.

Then, we used the inducible LCL-Z cell system to analyze several ligands for NK cells during EBV latency and upon switch to lytic replication, with a special focus on those molecules that have been controversially implicated in EBV-NK cell interaction in previous studies. Specifically, expression of NKG2DLs (MICA/B and ULBP1–4), DNAM-1Ls (CD112 and CD155), HLA-A, -B, and -C, HLA-E, and CD48 was measured by flow cytometry in both BZLF1^−^ and BZLF1^+^ LCL-Z cells after exposure to HT. **Figure 2A** shows representative results illustrating that, among NKG2DLs, MICB and, to a lesser extent, ULBP1, ULBP2, and ULBP4 were expressed on BZLF1^−^ cells in LCL-Z as well as in control non-transduced LCL cultures, while DNAM-1Ls were absent. This pattern was maintained in BZLF1^+^ LCL-Z cells with the exception of MICB expression that was significantly increased in terms of positive cells (18 ± 4 vs. 24 ± 5% MICB^+^, mean ± SEM) as well as MFI (1,400 ± 277 vs. 1,880 ± 300 MICB MFI) and of a trend towards downregulation of ULBP2 by comparing with BZLF1^−^ counterparts in six independent cultures (**Figures 2A, B**). As to HLA molecules, HLA-A, -B, and -C levels were modestly reduced in BZLF1^+^ as compared with BZLF1^−^ cells in LCL-Z cultures (75% ± 6% vs. 70% ± 6% HLA-A, -B, and -C^+^ and 26,670 ± 9,990 vs. 17,550 ± 5,170 HLA-A, -B, and -C MFI), whereas HLA-E was drastically downregulated in BZLF1^+^(35% ± 4% vs. 8% ± 1% HLA-E^+^ and 3,540 ± 1,160 vs. 374 ± 89 HLA-E MFI). A partial loss of HLA-E was also evident in BZLF1^−^ cells of HT-induced LCL-Z cultures (as compared to BZLF1^−^ LCLs, **Figure 2A**), suggesting that HLA-E expression was being affected by EBV starting from the very early phases of latency exit, when BZLF1 amounts are too low for positive cell gating by flow-cytometry. On the other hand, CD48 was expressed by all cells to a level that did not vary between BZLF1^+^ and BZLF1^−^ cells (**Figures 2A, B**). Results are shown at 48 h post-treatment with HT, but phenotypic changes start earlier (24 h) and are maintained at 72 h (data not shown).

To further investigate the impact on NK cell ligands of lytic EBV replication, HT-exposed LCL-Z were labeled after permeabilization for NKG2DL or HLA molecules to measure their intracellular expression levels (**Figure 2B**, lower panels). Of note, the intracellular levels of MICB as well as of ULBP2 and ULBP4 were increased in BZLF1^+^ cells as compared with BZLF1^−^ cells, while expression of other ligands was very low/undetectable in both cell populations; moreover, a significant reduction of HLA-E but not HLA-A, -B, and -C levels within BZLF1^+^ cells was found (**Figure 2B**). Finally, since NKG2DLs can be regulated via proteolytic shedding at the cell membrane, the presence of soluble MICA, MICB, and ULBP2 (referred to as sMICA, sMICB, and sULBP2) was analyzed by ELISA using the cell culture medium of LCL-Z exposed or not to HT for 48 h, with or without a broad-spectrum matrix metalloproteinase inhibitor (MMPI). Results showed that, while sMICA and sULBP2 were barely detectable in all conditions, sMICB accumulated in an MMP-dependent manner in LCL-Z cultures and to a higher extent in cultures with lytic EBV replication (**Figure 2C**).

Overall, these data demonstrate that NKG2DLs, but not DNAM-1Ls, are modulated during EBV infection; specifically, cell-surface MICB is expressed and can be released in a soluble form during latency and to a higher extent in cells with lytic EBV replication, whereas other NKG2DLs are very low or absent at the cell membrane in all phases of the viral life cycle, though ULBP2 and ULBP4 accumulate within cells in the lytic stage. In addition, all cell-surface HLA molecules are downregulated in cells with lytic EBV replication, with a much stronger effect on HLA-E that nearly disappears as compared with a modest reduction of HLA-A, -B, and -C molecules.

### 3.3 EBV entry into the lytic phase sensitizes LCLs to NK cell-mediated killing

**Figure 3:**
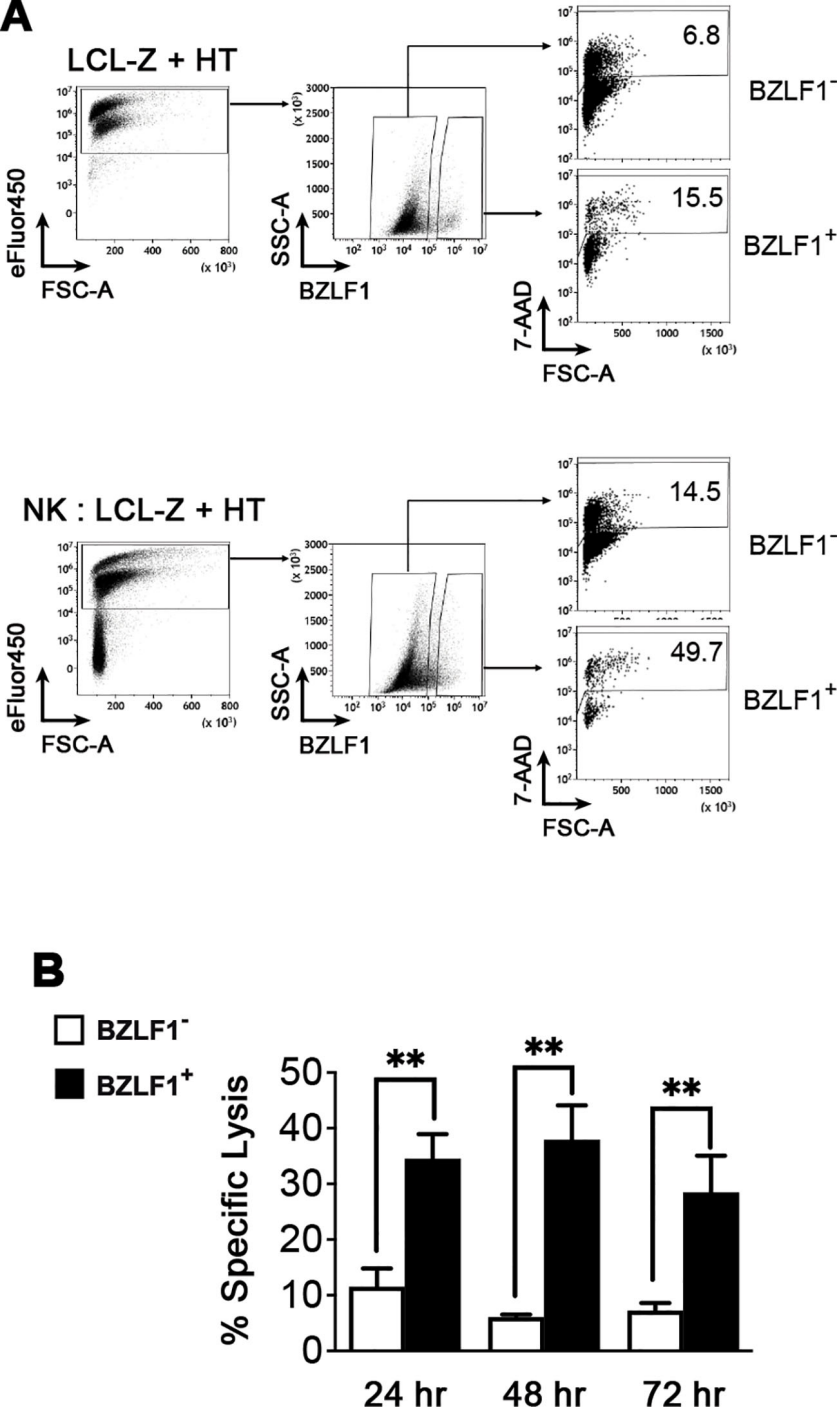
Sensitivity of LCLs with latent or lytic EBV infection to NK cell-mediated killing. A killing assay was set up in which LCL-Z targets stimulated with HT for 48 h were labeled with eFluor450 and then cultivated for 5 h with primary NK cells pre-activated with IL-2 at an E:T ratio of 2:1. The cultures were analyzed by flow cytometry to measure the frequency of dead (7-AAD^+^) cells among eFluor450^+^ targets gated as BZLF1^−^ and BZLF1^+^. **(A)** The gating strategy and percentages of dead cells are shown for a representative sample of LCL-Z + HT targets cultivated separately (top panels) or together with NK (bottom panels). **(B)** The percentage of NK cell-mediated specific lysis (mean ± SEM) was measured in eight different LCL-Z cell lines. **p < 0.01 by paired *t*-test.

Next, we used HT-stimulated LCL-Z to assess the susceptibility to NK cell-mediated killing of infected cells in both latent and lytic phases of the EBV life cycle. To this end, NK cells purified from healthy donors and activated with IL-2 served as effectors (E) in a 5-h flow cytometry-based cytotoxicity assay in which LCL-Z treated with HT for 24, 48, and 72 h were used as targets (T) at an E:T ratio of 2:1 (the gating strategy is shown in **Figure 3A**). We found that LCL-Z were rather poorly killed by NK cells during the latent phase at each post-stimulation time point, with a specific lysis of BZLF1^−^ cells corresponding to 11.5% ± 3.3%, 6.1% ± 0.4%, and 7.2% ± 1.4% (mean ± SEM; **Figure 3B**). Conversely, the EBV switch to lytic replication significantly enhanced NK cell-mediated clearance, resulting in 34.5% ± 4.4%, 37.9% ± 6.1%, and 28.5% ± 6.6% lysis of BZLF1^+^ cells (**Figure 3B**). In these experiments, LCL-Z targets were killed by allogeneic NK cells, but analogous results can be obtained in autologous settings (**Supplementary Figure S1**).

### 3.4 MICB upregulation on LCLs translates into NKG2D-mediated killing by NK cells

**Figure 4:**
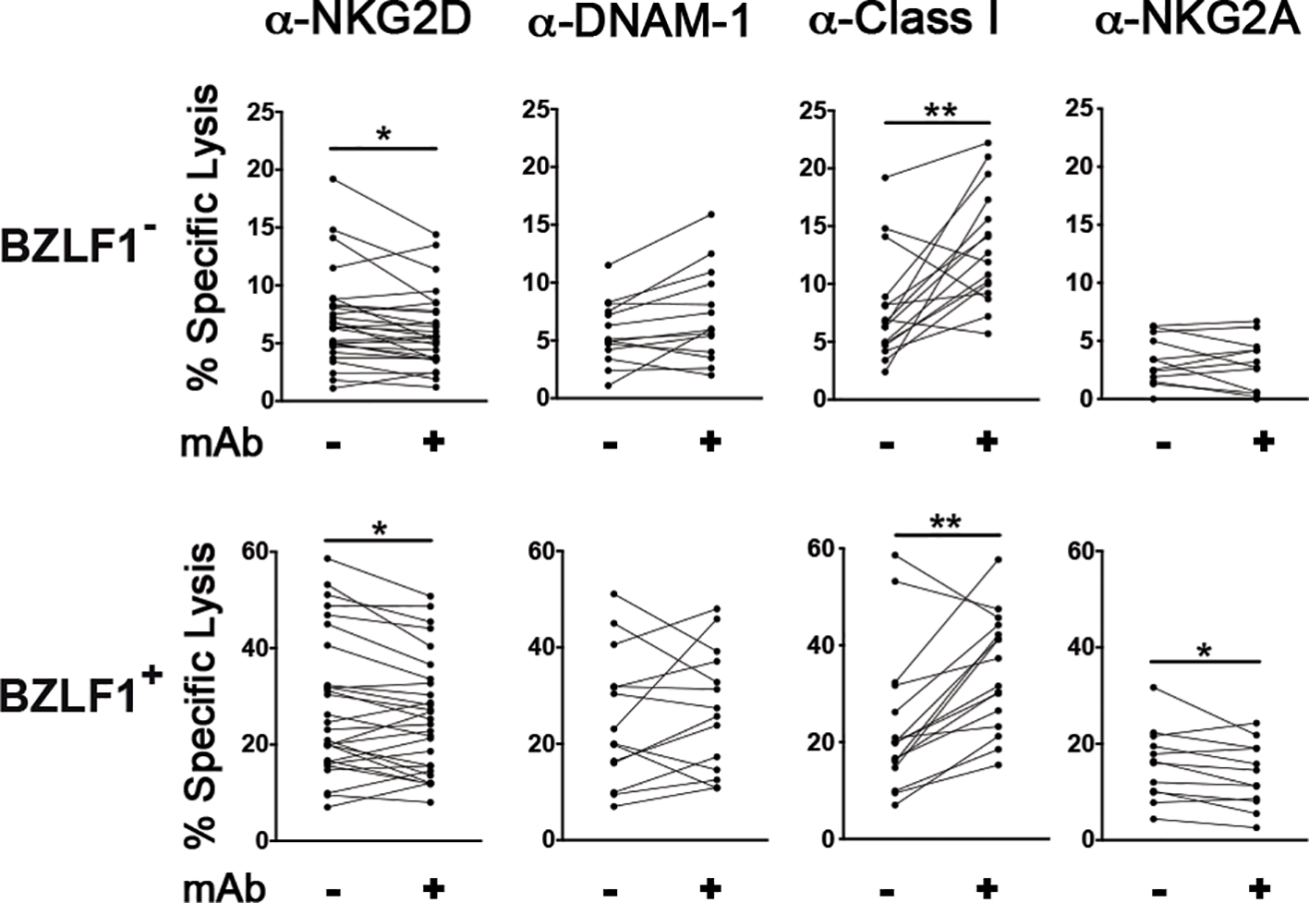
Effect of blocking antibodies on NK cell-mediated killing of LCL targets with latent or lytic EBV infection. Multiple HT-treated LCL-Z cell lines were employed as targets in NK lysis assays described in **Figure 1** in which antibodies blocking NKG2D (*n* = 28), DNAM-1 (*n* = 14), Class I molecules (*n* = 16), or NKG2A (*n* = 11) were used. The percentage of specific lysis was measured for both BZLF1^−^ and BZLF1^+^ targets. Statistics was carried out using paired Wilcoxon (panels with anti-HLA-Class I blocking antibody) and *t*-test for non-parametric and parametric distributions, respectively (*p < 0.05, **p < 0.01).

To examine the receptor/ligand pairs that mediate NK cell recognition and killing of LCLs with both latent and lytic EBV infection, lysis assays were performed in the presence of blocking antibodies directed against NKG2D or DNAM-1; in addition, pan-HLA class I specific antibody (IgM clone A6136) was added or not during the lysis assay. Blocking NKG2D but not DNAM-1 resulted in decreased NK cell-mediated killing of both BZLF1^−^ and BZLF1^+^ cells (**Figure 4**), consistent with MICB expression and the absence of DNAM-1Ls on these cell targets described herein (**Figures 2A, B**). On the other hand, the addition of anti-HLA class I antibody resulted in a higher cytotoxicity against not only BZLF1^−^ cells but also BZLF1^+^ cells, which display reduced HLA-A, -B, and -C and nearly absent HLA-E on their membrane (**Figures 2A, B**), indicating that residual expression of class I molecules on cells undergoing lytic EBV replication is sufficient for engaging inhibitory NK receptors, hence constraining activation of NK cell cytotoxicity (**Figure 4**). Finally, antibody-mediated blocking of NKG2A, an inhibitory receptor engaged by HLA-E that has been implicated in anti-EBV NK cell responses in previous *in vivo* and *in vitro* studies (24, 34, 35), had no effect on BZLF1^−^ cell killing but significantly reduced the lysis of BZLF1^+^ cells (**Figure 4**), suggesting a positive role for NKG2A in the capacity of NK cells to clear LCLs undergoing lytic EBV replication.

### 3.5 CD56^bright^ and NKG2A^+^KIR^+^CD56^dim^ NK cell subsets efficiently respond against LCLs with lytic EBV replication

**Figure 5:**
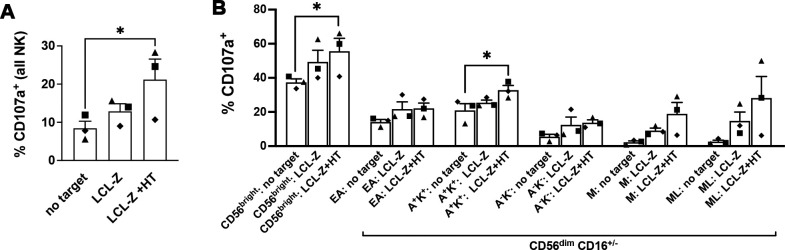
Frequencies of NK cells responding to LCLs with and without lytic EBV activation. Primary IL-2-stimulated NK cells were cultivated for 5 h alone or in the presence of autologous LCL-Z cells pre-treated or not with HT for 48 h, then analyzed by flow cytometry to measure expression of the CD107a degranulation marker. **(A)** Frequencies of CD107a^+^ NK cells are reported for the three culture conditions (no targets, with LCL-Z, or HT-treated LCL-Z). **(B)** Analysis of the distribution of gated CD107a^+^ NK cells into maturation subsets including CD56^bright^ and five CD56^dim^ subsets identified as NKG2A^+^KIR^−^ (Early-Differentiated, ED), NKG2A^+^KIR^+^ (A^+^K^+^), NKG2A^−^KIR^−^ (A^-^K^-^), NKG2A^−^KIR^+^CD57^+^ (Mature, M), and NKG2A^−^KIR^+^CD57^+^NKG2C^+^ (Memory-Like, ML). KIR labeling was performed combining three different antibodies recognizing KIR2DL1, KIR2DL2, and KIR3DL1. Bars show mean ± SEM of results obtained with three independent donors (each represented with a distinctive symbol). Statistics was performed using Friedman with Dunn’s multiple comparisons test (*p < 0.05).

The vast majority of IL-2-activated NK cells express NKG2D and hence have the potential to recognize and kill MICB^+^ LCL targets, whereas NKG2A is expressed by CD56^bright^ cells and subsets of CD56^dim^ cells corresponding to nearly half of the NK cell population (36); specifically, 44% ± 8% of NK cell samples tested in the present study were NKG2A^+^. Therefore, we thought to investigate which subset of NK cells reacted against LCLs with and without EBV reactivation by comparing the degranulation activity (in terms of CD107a expression) of IL-2-stimulated NK cells that have been cultivated for 5 h in medium alone (no targets) or with autologous LCL-Z pre-exposed or not to HT, and then gated into various maturation subsets by flow cytometry. The gating strategy separated CD56^bright^ cells and five subsets of CD56^dim^ cells further dissected in NKG2A^+^KIR^−^ (referred to as Early-Differentiated, ED), NKG2A^+^KIR^+^, NKG2A^−^KIR^−^, NKG2A^−^KIR^+^CD57^+^ (referred to as Mature, M), and NKG2A^−^KIR^+^CD57^+^NKG2C^+^ (Memory-Like, ML). Conventionally, CD56^bright^ cells comprise both CD16^−^ and CD16^+^ cells while CD56^dim^ cells are all CD16^+^; however, herein CD16^−^ cells were also included in the CD56^dim^ gate because we observed a partial loss of CD16 expression in CD56^dim^ cells after exposure to LCL-Z targets, likely consequent to CD16 shedding by activated NK cells (37), while other markers were largely maintained (**Supplementary Figure S2**). By analyzing the entire population of IL-2-stimulated NK cells (CD56^bright^ + CD56^dim^ cells), their basal degranulation modestly increased upon co-culture with LCL-Z cells but it was significantly higher if LCL-Z were previously exposed to HT (**Figure 5A**), in accord with killing data (**Figure 3**). Moreover, we found a general trend towards the higher frequency of CD107a^+^ in all gated subsets of NK cells challenged with LCLs as compared with NK cells cultured without targets (**Figure 5B**); of note, HT-exposed LCL-Z targets induced a statistically significant increment of CD56^bright^ and NKG2A^+^KIR^+^ cells within the CD107a^+^ population, indicating that these NK subsets are those that preferably respond against cells with lytic EBV replication. Results also showed a higher degranulation of the more differentiated M and ML subsets upon challenge with LCL-Z + HT targets, although not in one tested donor who also had the lowest overall degranulation activity (**Figure 5A**, diamond symbol) despite the unvarying NK cell phenotype and maturation pattern (**Supplementary Figure S3**).

## 4 Discussion

EBV is usually acquired in young age and persists throughout life in a state of latency without causing harm, yet various EBV-related LPD and malignant diseases can be incurred, particularly in patients with primary or secondary immunodeficiency. For EBV, there is no preventing/therapeutic vaccine or effective drug available, at present, and EBV-induced diseases are treated with conventional strategies, such as surgery and chemotherapy, which are often burdened by complications. On the other hand, rapidly evolving immunotherapies have recently offered novel opportunities, with some strategies already having proved their efficacy in EBV-related malignancies, including adoptive transfer of EBV-specific T cells and use of antibodies targeting B cells or blocking immune checkpoint signaling (38–41). Another promising approach to treat EBV^+^ malignancies rely on NK cell-based therapies that include modified NK cells with engineered receptors and activation/expansion of NK cell populations for adoptive transfer; of note, NK cells can outperform T cells in adoptive cell therapy because they have a general lower risk of graft-versus-host disease or other adverse events, and display effector functions that are not sensitive to escape mutations in T-cell antigens, which often occur in EBV (42, 43). Importantly, accumulating evidence has demonstrated that the induction of EBV-induced malignancies is not an exclusive prerogative of viral latency proteins as lytic factors also contribute to tumorigenesis (6, 44, 45); hence, therapeutic targeting of both latent and lytic phases may be essential.

In the present study, to investigate the interaction of NK cells with EBV throughout its life cycle, we generated multiple inducible LCL-Z cell lines that, in the absence of stimuli, typically resemble EBV-associated B-cell lymphomas with type III latency, but then, upon HT-mediated activation of recombinant BZLF1 protein, they rapidly enter into the lytic replication phase. The lytic EBV replication, for which no permissive cell system is currently available, is usually investigated in those few cells among LCL or BL cell cultures in which the virus is spontaneously reactivated or, more often, after treatment with inducing agents such as phorbol esters, calcium ionophores, histone deacetylase inhibitors, or Ig crosslinkers. Conversely, in the LCL-Z cell system, the switch to the lytic replication phase occurs efficiently with a physiologic, i.e., BZLF1-initiated, modality, hence avoiding the use of EBV-reactivating drugs that profoundly alter gene expression of the host cell. By analyzing LCL-Z cells with and without lytic reactivation, we demonstrated that, among NKG2DLs and DNAM-1Ls, only MICB is significantly expressed at the cellular membrane during latency and to a higher level in cells that entered the lytic cycle, which translates into killing by NK cells in a manner that is dependent on the cognate NKG2D receptor. In addition, we detected increased intracellular levels of MICB as well as of ULBP2 and ULBP4 within lytic cells and accumulation in LCL-Z cultures of sMICB that was higher in cultures with lytic EBV replication. Apparently, infection with EBV results in up-modulation of NKG2DLs, particularly MICB, which is a common cellular defense against viruses mediated by activation of stress response pathways (46) while, analogously to other viruses, EBV has evolved the capacity to contrast cell-surface NKG2DL expression in order to avoid immune recognition, specifically retaining MICB, ULBP2, and ULBP4 into intracellular compartments and inducing the release of sMICB from the cell membrane through the activity of viral factors that are currently unknown. Our results are in line with some previous studies showing that latently infected LCLs express MICB along with, whenever tested, low levels of MICA and ULBP4, and they are susceptible to NKG2D-dependent killing by NK cells as well as EBV-specific CD8 T-cell clones or γδ T cells (29, 30, 47). Also in accordance with our data, it was shown that the LMP2A latency protein of EBV, which activates signaling pathways to promote the proliferation of latently infected cells, induces MICA and MICB expression at the transcriptional level, while, on the other hand, reducing the cell-surface expression of both MIC proteins and ULBP4 through mechanisms that have not been identified as yet (30, 48). Additional lines of evidence indicating that EBV has developed means to contrast NKG2DL expression was provided by studies on viral ncRNAs showing that miR-BART2-5p and miR-BART7 impair translation of MICB and MICA mRNA, respectively (29, 49). These EBV activities have important functional consequences for NK cell responses, since inhibition of miR-BART2-5p in latent LCLs resulted in MICB upregulation and enhanced killing by NK cells (29), whereas overexpression of miR-BART7 in NPC lowered rates of NK cell-mediated lysis (49). Finally, studies in patients with mutated *MAGT1* (referred to as “X-linked MAGT1 deficiency with increased susceptibility to EBV-infection and N-linked glycosylation defect”, XMEN) clearly pointed at a key role of the NKG2D/NKG2DL axis in the control of EBV-related diseases, which are the main cause of severe morbidity in these patients (50); specifically, because of the absence of MAGT1-mediated glycosylation, NKG2D is strongly downregulated on patients’ NK and CD8 T cells that, as a consequence, are poorly cytotoxic *in vitro* against autologous NKG2DL^+^ (i.e., positively stained by means of a recombinant NKG2D-Fc molecule) LCLs, unless NKG2D expression is previously restored. It should be mentioned, though, that some previous lines of evidence are discordant with our results; in particular, one study showed that cells with lytic EBV replication do not express various tested NKG2DLs (i.e., MICA, MICB, and ULBP2) or DNAM-1Ls (CD122 and CD155) and are killed by NK cells in a manner that is independent of NKG2D but requires DNAM-1, implying the engagement with a hypothetical DNAM-1L yet to be identified (31). We suggest that discrepancies are linked to differences in the experimental system employed, consisting in challenging an NK cell line (NKL) with lytic BL cells (AKBM treated with anti-IgG) or spontaneously reactivated LCLs.

To evade CD8 T-cell responses, EBV has developed a complex strategy targeting the antigen presentation machinery (APM) and HLA class I molecules that, on the other hand, may activate NK cells through the lack of inhibitory KIR/HLA class I interactions according to the “missing self” model of NK cell lysis (8). Specifically, by means of EBV activation in BL cell lines, transduction of individual EBV genes into EBV^−^ tumor cell lines, or EBV infection of primary B cells, it has been demonstrated that expression of AMP and HLA class I is stimulated by the latent LMP1 protein while the opposite effect is exerted by various lytic proteins and ncRNAs, with cell-surface HLA-A, -B, and -C molecules being drastically downregulated following entry into the lytic phase [reviewed in Ref (51).]. In the present study, we found that LCL and unstimulated LCL-Z cells express high HLA-A, -B, and -C levels in agreement with previous reports (24, 52), which reflects the nature of activated blasts and expression of LMP1; once entered into the lytic phase, HLA-A, -B, and -C levels were reduced both at the cell surface and intracellularly in a statistically significant manner, yet to a very modest extent. In accord with these results, antibody-mediated block of HLA molecules resulted in higher NK cell-mediated killing of both BZLF1^−^ and BZLF1^+^ targets, indicating that LCLs are protected from cytolysis by the interaction between HLA ligands with inhibitory KIRs on effector cells throughout the viral life cycle. Therefore, partial reduction of HLA-A, -B, and -C during lytic EBV replication is important for the virus to escape CD8 T-cell recognition, particularly via the downregulation of specific HLA-B molecules that present immunodominant EBV peptide during primary B-cell infection (53), but apparently is not sufficient to trigger NK cell activation. It should be noted, though, that NK cell inhibition via KIR/HLA-A, -B, and -C interactions shall be confirmed with EBV^+^ targets other than LCLs, for instance, EBV-associated tumors in which HLA class I molecules can be downregulated also by cellular oncogenic factors, as shown for c-Myc in the context of NPC (54).

Moreover, our results show an important role for the NKG2A/HLA-E interaction in the capacity of NK cells to kill LCL targets. In line with some previous studies in BL cells (26, 55), we found that latent LCLs efficiently express HLA-E, non-classical HLA class I molecules that present peptides derived from leader sequences of HLA-A, -B, -C, and -G molecules; then, upon EBV switch to lytic replication, HLA-E is drastically downregulated. HLA-E/peptide complexes normally suppress NK cell function by binding to CD94 in the heterodimeric NKG2A/CD94 receptor, which results in NKG2A-mediated inhibitory signaling; this important immune checkpoint can be targeted by blocking antibodies that are currently in clinical development to treat HLA-E^+^ tumors (56). Unexpectedly, we found that blocking of NKG2A via a specific antibody had no effect on the capacity of NK cells to kill latent LCLs, notwithstanding their HLA-E expression, and clearance of lytic BZLF1^+^ cells that are nearly devoid of HLA-E was rather decreased. This apparent discrepancy, however, can be explained by considering some peculiar aspects of HLA-E expression in EBV-infected cells as well as of NKG2A biology. First, peptides derived from both latent and lytic EBV proteins have been shown to associate with HLA-E molecules and prevent NKG2A triggering, thus unleashing the cytotoxic responses of NKG2A^+^ NK cells (57, 58), a phenomenon that may account for the lack of NKG2A-mediated inhibition of LCL killing in our cell system. Second, while KIR-mediated inhibition of NK cells follows a linear relationship with the levels of HLA-A, -B, and -C on targets, high HLA-E levels are required for NKG2A to inhibit activated NK cells (59). Moreover, inhibitory signals delivered by KIRs and NKG2A are needed for NK cells to become fully functional through a process referred to as “education” (also “arming” or “licensing”) in which NKG2A has the strongest impact (60). Accordingly, it was reported that tumors with low HLA-E levels are killed more efficiently by NKG2A^+^ than NKG2A^−^ NK cells and independently of KIR/HLA-A, -B, and -C interactions (61), indicating that better education by NKG2A can prevail on its inhibitory signaling, at least when HLA-E is poorly expressed on target cells. Therefore, we suggest that NKG2A cannot inhibit NK cell activation against LCLs due to the association of decoy viral peptides to HLA-E that impair engagement of NKG2A throughout the EBV life cycle as well as to insufficient HLA-E expression during the lytic phase. On the other hand, NKG2A identifies the population of NK cells that react against LCLs with lytic EBV replication, since the use of anti-NKG2A antibody reduced killing of BZLF1^+^ cells in our lysis assay. We assume that, in the absence of NKG2A/HLA-E association, engagement with anti-NKG2A antibody may trigger receptor-mediated inhibitory signals and, ultimately, inhibit cytotoxicity of NKG2A^+^ NK cells against BZLF1^+^ targets. Accordingly, by investigating the phenotype of NK cells that degranulated against LCL-Z cells with or without EBV reactivation, we showed that NKG2A^+^ NK cell subsets, specifically CD56^bright^ and NKG2A^+^KIR^+^CD56^dim^ cells, are those that better respond against cells with lytic EBV replication. Our results are largely in agreement with previous *in vivo* and *in vitro* evidence, including recruitment in the tonsils of EBV^+^ individuals of a CD56^bright^ NKG2A^+^ NK cell subset with a strong IFN-γ-mediated capacity to suppress EBV replication *in vitro* (7) and expansion during IM of circulating NKG2A^+^CD56^dim^ NK cells that are proficiently activated when challenged with lytic BL cells (23, 24). In addition, *in vitro* experiments using IL-2-activated NK cells as effectors showed that CD56^dim^NKG2A^+^ cells are preferentially activated against autologous LCLs, although targets with lytic EBV replication were not tested (34). When in-depth analysis of NKG2A^+^CD56^dim^ cells reacting *in vitro* against EBV^+^ targets has been performed, this showed not only high expression of NKG2D and loss of CD16 in line with our results (34, 35, 62), but also lack of KIR expression, which is at odds with our finding that NKG2A^+^KIR^+^CD56^dim^ cells are those that better respond against lytic LCLs. However, we think that the present study differs from others in that it is the first one to investigate the overall distribution into maturation subsets of NK cells that have reacted against autologous LCLs with either latent or lytic EBV replication. Interestingly, the simultaneous presence of NKG2A and KIRs is indicative of highly educated CD56^dim^ cells provided with full responsiveness to target cells (63).

It may seem counterintuitive that EBV has evolved means to impair NKG2A-mediated inhibition of NK cells and elicit the antiviral effector function of NKG2A^+^ NK cells. However, two distinct viruses, HIV-1 and SARS-CoV-2, also encode for HLA-E binding peptides that abrogate inhibition of NKG2A^+^ NK cells, so that infected cell targets (CD4 T and epithelial lung cells, respectively) are killed *in vitro* more efficiently by NKG2A^+^ NK cells (both CD56^bright^ and NKG2A^+^ CD56^dim^) than NKG2A^−^ counterparts (64, 65). It is plausible that viral HLA-E binding peptides correspond to conserved sequences critical for the replication fitness of EBV, HIV-1, and SARS-CoV-2 viruses that, on the other hand, have evolved other immune evasion strategies (e.g., downregulation of ligands for activating receptors) to counterbalance missing-self activation of NKG2A^+^ NK cells.

This study has limitations because host genetic factors that fine-tune NK cell functionality have not been considered, including *HLA* polymorphisms that influence HLA-E stability and education of NKG2A^+^ NK cells (66) and *HLA* alleles and *KIR* haplotypes associated with EBV infection and disease (67). Another aspect that deserves to be further investigated is the capacity to clear EBV^+^ targets of adaptive NK cells that express the inhibitory CD94/NKG2C receptor for HLA-E and display superior effector functions in the context of HCMV infection (15). In general, all our results should be confirmed in clinical samples. In particular, it should be investigated whether MICB is upregulated in various EBV-associated tumors and eventually accumulate in a soluble form in patients’ plasma. Likewise, the expression and function of NKG2D in NK cells of patients with EBV-related disease require investigation since chronic binding to cell-surface or soluble NKG2DLs can lead to receptor “detuning” and overall desensitization of NK cells (68). These studies will shed light on the potential of the NKG2D/MICB axis as a therapeutic target in EBV-driven malignancies. At present, several bioengineered molecules harnessing the NKG2D pathway against cancer have been developed such as antibodies that block MIC protein shedding, the extracellular NKG2D domain fused to immune activating components, and the NKG2D-based chimeric antigen receptor (CAR.NKG2D) [reviewed in Ref (69).]. Moreover, we speculate that the development of allogenic HLA mismatched NK cells with the NKG2A^+^KIR^+^ phenotype and enhanced expression of NKG2D may harbor great potential for adoptive cell therapies in patients with EBV^+^ malignancies. Finally, the efficacy of NK cell-based therapies may be further increased by the combination with the “lytic induction” approach (70), which implies reactivation of EBV with histone deacetylase inhibitors or other epigenetic drugs that are well-known for their potent capacity to induce NKG2DL expression among various biological effects (71).

## Data Availability

The raw data supporting the conclusions of this article will be made available by the authors, without undue reservation.
