# Supplementary material for: Entry into the lytic cycle exposes EBV-infected cells to NK cell killing via upregulation of the MICB ligand for NKG2D and activation of the CD56bright and NKG2A+KIR+CD56dim subsets
Source: Front Immunol. 2024 Nov 29;15:1467304. doi: 10.3389/fimmu.2024.1467304 (PMC11638013; doi:10.3389/fimmu.2024.1467304)
Supplement: Supplementary file 1 [file DataSheet1.pdf]

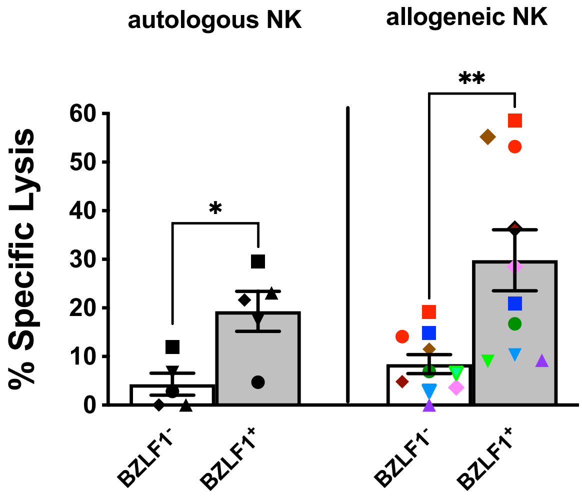

Supplementary Figure S1. Comparison of killing assays in autologous and allogeneic settings. Five distinct LCL-Z cell lines (identified with different symbol shapes) treated with HT for 48 h were used as targets in lysis assays performed with autologous NK cells (filled black symbols) or with NK cells derived from different donors (colored symbols). Bars show mean ± SEM percentages of specific lysis of BZLF1^{-} and BZLF1^{+} targets. **P* < 0.05, ***P* < 0.01 by paired *t*-test.

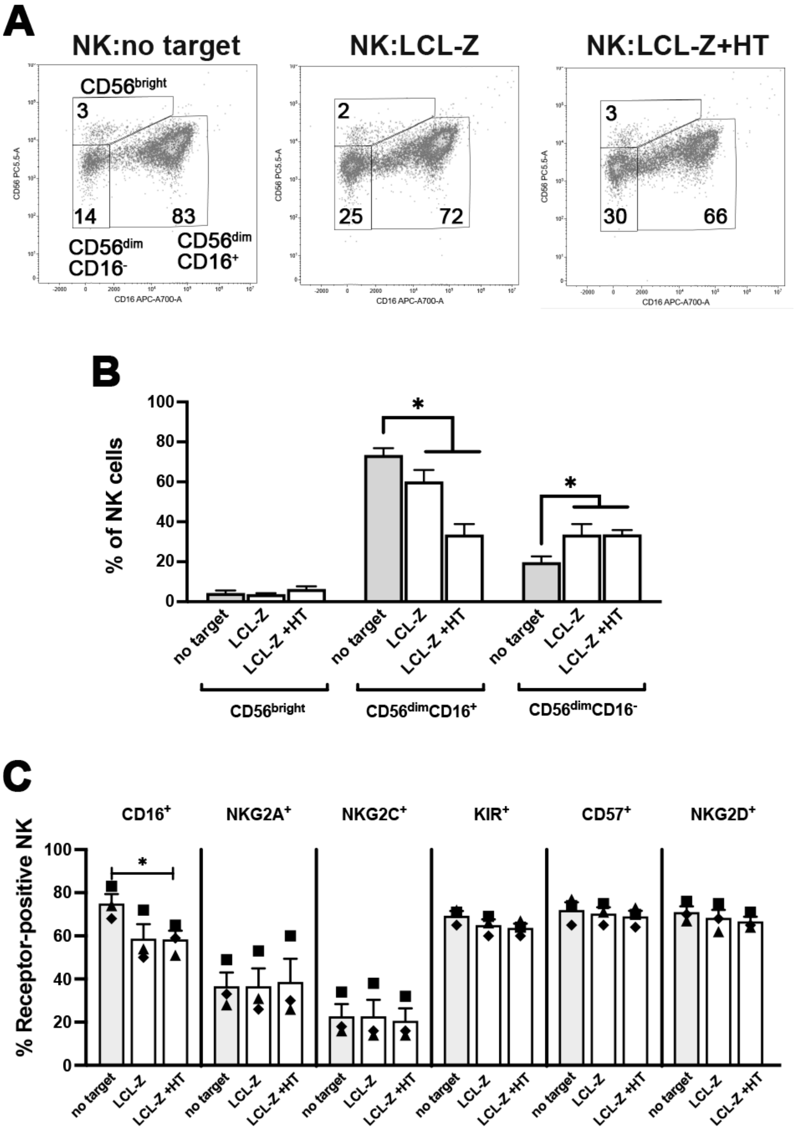

Supplementary Figure S2. Analysis of NK cell subsets frequency upon IL-2 stimulation for 16 hr followed by 4 hr culture alone or in the presence of autologous LCL-Z targets that were pre-treated or not with HT for 48 hr as described in Figure 5. Representative dot plots **(A)** and mean ± SEM of 3 independent donors **(B)** show the frequencies in the three culture conditions of NK cell subsets gated as $CD56^{bright}$, $CD56^{dim}CD16^{+}$ and $CD56^{dim}CD16^{-}$ cells, with the latter subset being nearly absent in freshly isolated NK cells (data not shown) but present in IL-2 stimulated cells (no targets) and increased upon challenge with targets (LCL-Z and LCL-Z + HT). **(C)** Bars show mean ± SEM percentage of cells positive for the expression of various markers (CD16, NKG2A, NKG2C, KIR, CD57, NKG2D) in the entire NK cell populations following the three culture conditions. Statistics was performed using One Way Anova with Tukey's multiple comparison test (**P* < 0.05).

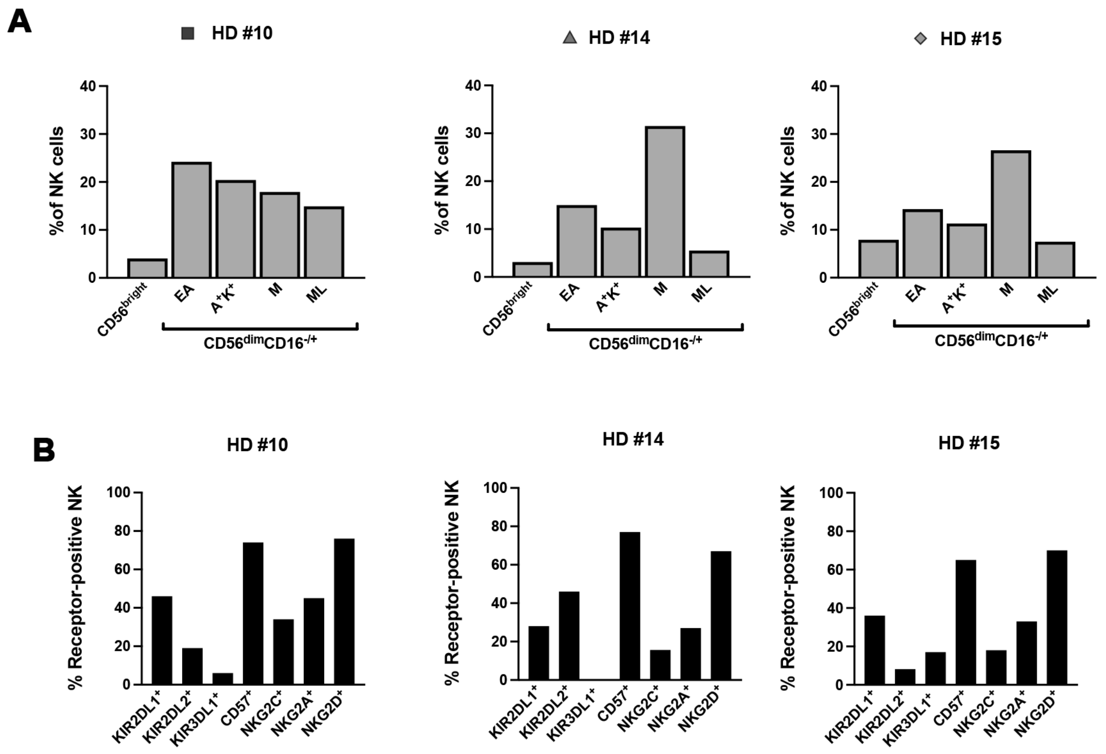

Supplementary Figure S3. Individual analyses of NK cells derived from three healthy donors (HD #10, HD #14, and HD #15) and employed in the experiment shown in Figure 5 (square, triangle, and diamond symbols, respectively) are shown. Distribution of maturation subsets **(A)** and percentage of cells expressing various markers (KIR2DL1, KIR2DL2, KIR3DL1, CD57, NKG2C, NKG2A, NKG2D) **(B)** are reported.
